# Is interleukin-18 associated with polycystic ovary syndrome?

**DOI:** 10.1186/1477-7827-9-7

**Published:** 2011-01-18

**Authors:** Yan Yang, Jie Qiao, Rong Li, Mei-Zhi Li

**Affiliations:** 1 Department of Obstetrics and Gynecology, Peking University Third Hospital, Beijing, China

## Abstract

**Background:**

Recent research show that polycystic ovary syndrome (PCOS) may have an association with low-grade chronic inflammation, IL-18 is considered as a strong risk marker of inflammation.

**Methods:**

To investigate serum IL-18 concentrations in PCOS patients and focus on its relationship between obesity and insulin resistance (IR). Sixty consecutive women with PCOS and thirty controls were recruited. Serum level of IL-18 and fasting blood glucose, fasting insulin, follicle-stimulating hormone (FSH), luteinizing hormone (LH) and testosterone (T) were measured.

**Results:**

Serum levels of IL-18 was significantly higher in the PCOS group than in the control group. Serum level of IL-18 was higher in the PCOS group with IR than in the PCOS group without IR. Serum level of IL-18 was higher in obese PCOS patients than in lean PCOS patients. Serum level of IL-18 was higher in lean PCOS patients than in the lean control group. Serum level of IL-18 in the PCOS group was positively related to BMI, IR index and T.

**Conclusion:**

IL-18 level was increased in PCOS patients, and correlated with insulin resistance, obesity and hyperandrogenism.

## Background

Polycystic ovary syndrome (PCOS) is a common and complex endocrine disorder of women in their reproductive years, with prevalence between 5% and 10% [1,2]. PCOS is characterized by chronic anovulation, hyperandrogenism, and insulin resistance (IR) [3]. Additionally, PCOS is often associated with obesity and a subsequent increased risk for type 2 diabetes [4-6]. Women with PCOS may have chronic low-level inflammation. Kelly et al [7] have reported increased serum C-reactive protein (CRP) level in PCOS patients. Amato et al [8] found that serum and follicular fluid interleukin-6 (IL-6) and tumor necrosis factor-alpha (TNF-α) values were higher in PCOS women than in controls. Interleukin-18 (IL-18) is a proinflammatory cytokine that induces the production of TNF-α [9], which in turn promotes the synthesis of IL-6 [10], and IL-6 regulates the synthesis of CRP in the liver [11]. Like IL-6 and CRP, IL-18 is considered a strong risk marker of cardiovascular death [12]. In order to investigate the possible roles of IL-18 in the pathogenesis of PCOS, we studied the serum level of IL-18 in PCOS patients, as well as it's correlation with IR, obesity and hyperandrogenism.

## Methods

### Patients and control group

A total of 60 patients with PCOS were included in this prospective, case-controlled study. We analyzed data from women with PCOS who visited the Department of Obstetrics and Gynecology, Division of Reproductive Center, Peking University Third Hospital, Beijing, from October 2006 to January 2007. The diagnosis of PCOS was based on the 2003 Rotterdam ESHRE/ASRM criteria: (1) oligo- and/or anovulation; (2) clinical and/or biochemical signs of hyperandrogenism (patients presented with hirsute, acne or alopecia, and/or increased circulating levels of testosterone; (3) polycystic ovaries (ovarian morphology was assessed using transvaginal ultrasound), and exclusion of other aetiologies (congenital adrenal hyperplasia, androgen-secreting tumors, Cushing's syndrome)[13-15]. Women who had received any hormonal treatment or insulin-lowering agent during the last 3 months were excluded from the study.

The control group consisted of 30 subjects, the controls were selected from women attending the clinic on account of male azoospermia. All controls had regular menstrual cycle and normal androgen level and were taking no medication. All patients were Chinese and had not taken hormonal medications, including contraceptive pills, for the last 6 months. None of the patients had clinical evidence (history or examination) of recent or ongoing infection. Institutional Review Board approval was obtained for this study and patients' consent was obtained from all women prior to inclusion in the study.

Firstly, the PCOS patients were divided into two sub-groups PCOS IR group and PCOS without IR group, insulin resistance was judged by using the homeostatic model index (HOMA-IR), and 2.69 was selected as a cutoff point[16,17]. Thirty cases of PCOS with insulin resistance (HOMA-IR ≥2.69), thirty cases of PCOS without insulin resistance (HOMA-IR < 2.69).

Secondly, the PCOS patients were divided into obese PCOS patients and lean PCOS patients. The diagnosis of obesity was based on the criteria of Asia-Oceania[18], which was defined by a BMI greater than or equal to 25 kg/m^2^. Twenty-nine cases of obese PCOS (BMI ≥2.5 kg/m^2^) and thirty-one cases of lean PCOS (BMI < 2.5 kg/m^2^).

Blood samples were obtained between days 2 and 3 of the menstrual cycle. In patients with amenorrhea, bleeding was induced by progestogen and blood samples were taken thereafter. If no bleeding occurred, blood samples were taken after excluding pregnancy by a commercially available pregnancy test. Blood was taken from the antecubital vein after a 12-hours overnight fasting. Samples were immediately centrifuged, and serum was separated and frozen at -20°C until assayed.

Body Mass Index (BMI) was calculated as follows: weight (kilograms)/height^2 ^(meters). Homeostasis model assessment index for IR (HOMA-IR) was calculated as follows: fasting insulin (mIU/l) × fasting glucose (mmol/L)/22.5.

### IL-18 enzyme-linked immunosorbent assay (ELISA)

Serum was separated and frozen at -20°C until assayed. Fasting insulin (FIN), follicle- stimulating hormone (FSH), luteinizing hormone (LH) and testosterone (T) levels were determined by chemiluminescence-immunoassay (CLIA) and fasting glucose (FBG) levels were detected using the glucose oxidase method. Serum IL-18 levels were measured using ELISA (Human IL-18 ELISA Kit, Bender MedSystems. Ltd., Vienna, Austria) with a lower limit of detection of 9.2 pg/mL and mean intra- and interassay coefficients of variation of 6.5 and 8.1%, respectively.

Statistical analyses were performed using SPSS 11.5 (SPSS Inc., Chicago, IL), with P < 0.05 being considered statistically significant. We analyzed the difference of serous components in three groups by correlation analysis, and the correlation between the levels of these serous components by linear regression analysis.

## Results

The mean level of IL-18 in PCOS IR group was 243.1 pg/mL (S.D. = 64.2), in the PCOS without IR group the mean level was 174.3 pg/mL (S.D. = 66.8) and in the control group the mean level was 122.4 pg/mL (S.D. = 40.2). Serum IL-18 concentrations were increased in PCOS patients irrespective of the presence or absence of IR, and PCOS women with IR presented with increased IL-18 level than PCOS without IR. PCOS patients presented with increased LH, T, HOMA-IR and LH/FSH levels, especially in PCOS group with IR, and there was a statistically significant difference (P < 0.05) (Table 1).

**Table 1:** Comparison of plasma biochemical variables in PCOS with insulin resistance(IR) group, PCOS without insulin resistance group and controls

	PCOS with IR (n = 30)	PCOS without IR (n = 30)	Controls (n = 30)
Age(year)	28.4 ± 3.8	28.2 ± 3.4	28.4 ± 3.9
BMI(kg/m^2^)	27.0 ± 3.8	22.5 ± 3.7	21.3 ± 2.3
FBG(mmol/L)	5.1 ± 0.7^Δ^	4.7 ± 0.5	5.0 ± 0.6
FINS(uIU/ml)	20.5 ± 3.1*^Δ^	7.1 ± 2.9	6.2 ± 3.1
FSH(IU/L)	6.1 ± 1.5*	6.2 ± 1.7*	6.9 ± 1.2
LH(IU/L)	11.5 ± 4.7*	9.7 ± 4.8*	4.5 ± 1.9
T(nmol/L)	1.9 ± 0.7*^Δ^	1.6 ± 0.5*	1.1 ± 0.5
Homa IR	4.6 ± 1.7*^Δ^	1.5 ± 0.6	1.4 ± 0.8
LH/FSH	1.9 ± 0.8*^Δ^	1.4 ± 0.5*	0.7 ± 0.3
IL18 (pg/ml)	243.1 ± 64.2*^Δ^	174.3 ± 66.8*	122.4 ± 40.2

Values are given as means ± SD.

*Compare with controls, P < 0.05; ^Δ^compared with PCOS patients without IR, P < 0.05.

The mean level of IL-18 in obese PCOS women was 247.5 pg/mL (S.D. = 63.6), in the lean PCOS women the mean level was 172.5 pg/mL (S.D. = 64.0) and in control group the mean level was 122.5 pg/mL (S.D. = 40.2). Serum IL-18 concentration was increased in PCOS patients irrespective of the presence or absence of obese, moreover, the obese PCOS patients presented with higher IL-18 level than lean PCOS patients (*P *= 0.000). Obese PCOS women presented with increased fasting insulin, LH, T, HOMA-IR and LH/FSH, compared with lean PCOS women. The mean level of IL-18 in lean controls was 118.6 pg/mL (S.D. = 34.9). Serum IL-18 concentration was higher in lean PCOS patients than in controls (*P *= 0.000). Serum level of fasting insulin, LH, T, HOMA-IR and LH/FSH were higher in PCOS women than in controls with normal BMI, and there was a statistically significant difference (P < 0.05) (Table 2).

**Table 2:** Comparison of serum biochemical variables and inflammatory markers between obese PCOS women and lean PCOS women and lean controls

	Obese PCOS (n = 29)	Lean PCOS (n = 31)	Lean controls (n = 29)
Age (year)	28.3 ± 2.4	28.1 ± 3.0	28.0 ± 2.7
BMI(kg/m^2^)	28.0 ± 2.8	22.1 ± 3.4	21.0 ± 2.0
FBG (mmol/L)	5.1 ± 0.7*	4.9 ± 0.5^Δ^	5.0 ± 0.5
FINS (uIU/ml)	16.8 ± 8.9*	11.0 ± 7.6^Δ^	6.1 ± 3.1
FSH (IU/L)	6.3 ± 1.4	6.0 ± 1.7^Δ^	7.0 ± 1.2
LH (IU/L)	12.3 ± 4.8*	9.1 ± 4.3^Δ^	4.6 ± 2.0
T (nmol/L)	2.0 ± 0.7*	1.5 ± 0.5^Δ^	1.1 ± 0.5
Homa IR	4.1 ± 2.1*	2.1 ± 1.4^Δ^	1.4 ± 0.7
LH/FSH	1.9 ± 0.6*	1.4 ± 0.6^ΔΔ^	0.7 ± 0.3
IL18 (pg/ml)	247.5 ± 63.6*	172.5 ± 64.0	118.6 ± 34.9

Values are given as means ± SD.

*Compare with lean PCOS, P < 0.05; ^Δ^compared with lean controls P < 0.05.

**Figure 1:**
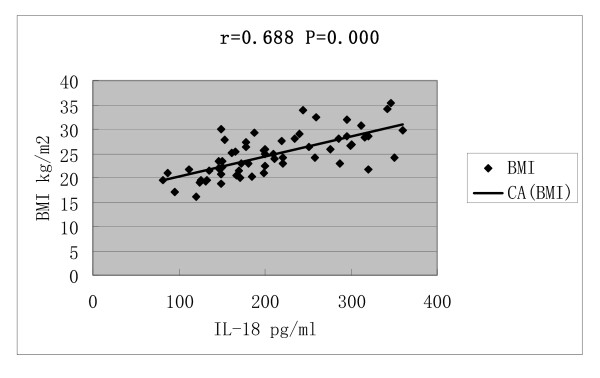
**Correlations of serum IL-18 concentrations with body mass index(BMI)**.

**Figure 2:**
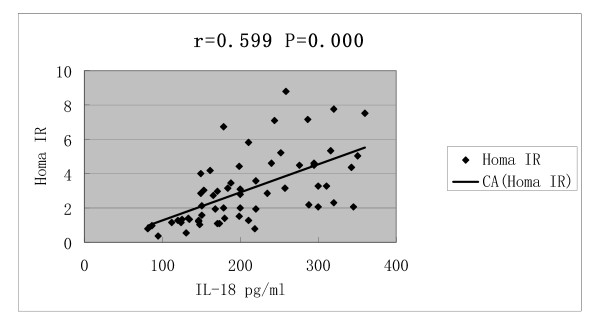
**Correlations of serum IL-18 concentrations with Homa IR**.

**Figure 3:**
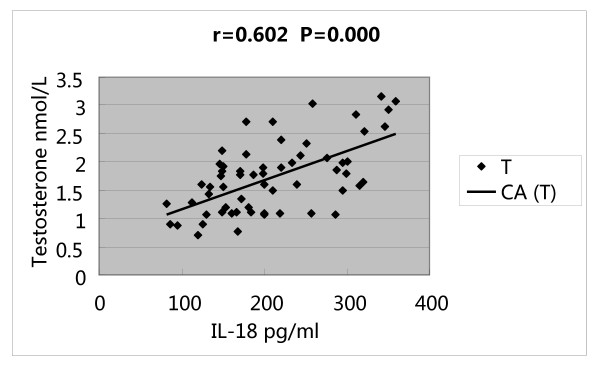
**Correlations of serum IL-18 concentrations with testosterone(T)**.

**Figure 4:**
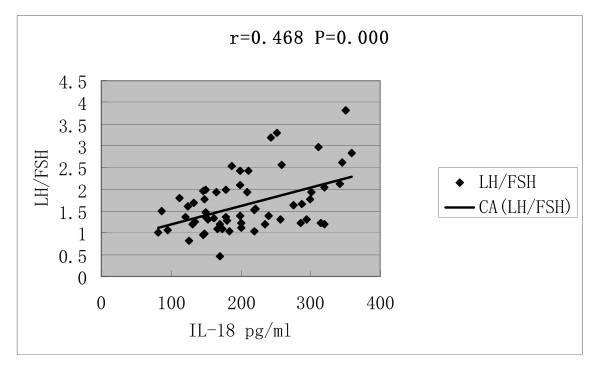
**Correlations of serum IL-18 concentrations with LH/FSH**.

Serum IL-18 concentrations was correlated with BMI (r = 0.688, P = 0.000), HOMA-IR (r = 0.599, P = 0.000), T (r = 0.602, P = 0.000) and LH/FSH (r = 0.468, P = 0.000) (Figures 1, 2, 3 and 4).

Linear regression analysis, including serum IL-18 concentration as the dependent variable and stepwise (probability of F to enter ≤ 0.05; probability of F to remove ≥ 0.10) introduction of BMI, HOMA-IR, T levels and LH/FSH as independent variables, showed that HOMA-IR, BMI and T determined 54.7% serum IL-18 concentration, [IL-18 = - 34.708 + 9.022 × HOMA-IR + 6.692 × BMI (kg/m^2^) + 29.066 × T (nmol/L); R^2 ^= 0.547; F = 24.745; *P *= 0.000], whereas LH/FSH was removed from the regression equation. Therefore, serum IL-18 levels appear to be determined by BMI, T and IR, and serum level of T seemed to be the most important influencing factor.

## Discussion

IL-18 was first described as an IFN-γ inducing factor, and has multiple functions which include of the synthesis of IFN-γ by T cells and NK cells, promotion of Th1-type immune response, augmentation of proliferative response and cytokine production of activated T cells. Meanwhile, IL-18 leads to activities against pathogens, by activating effector cells involved in the cellular interactions that occur during inflammation [19,20]. In our study, serum IL-18 concentration was increased in PCOS patients irrespective of the presence or absence of IR and obesity. Therefore, the PCOS disease per se has correlation with IL-18, not dependent on IR and obesity.

In our study, PCOS patients complicated with IR and obesity presented with increased IL-18 level, and serum IL-18 concentration had positive correlation with IR and BMI, it seemed that IR and obesity may accelerate the increase of serum IL18 level. The correlation of IL-18 level with BMI observed in our study also suggested that IL-18 might be produced by adipose tissue. In 2004, Escobar-Morreale et al [21] had reported that PCOS and obesity induced an increase in serum IL-18 levels, which was also associated with several indexes of global and visceral adiposity and with IR. IL-18 secretion by adipose tissue has been explored or reported elsewhere, but the mechanism needs to be addressed by future studies.

In our series, IL-18 concentration had positive correlation with T, and linear regression analysis showed that serum IL-18 concentration was determined by T, BMI and HOMA IR, but especially by T. Moreover, it seemed that hyperandrogenism was correlated with serum level of IL-18, and it is a crucial factor of increased level of IL-18, which might be the reason for PCOS patients who without insulin resistance and obesity have elevated IL-18, but the mechanism of it has not been clear, it need future study.

Mounting evidence suggests that IL-18 is involved in the pathogenesis of obstertrical disease and metabolic syndrome. Recently study showed that increased serum level of IL-18 was observed in preeclampsia [22], premature rupture of membranes, acute fatty liver of pregnancy and fetal growth restriction [23]. So IL-18 maybe paly an important role in pregnancy. Futhermore, some recent reports showed that IL18 might be correlated with atherosclerosis [24] and served as a cardiovascular risk marker [25,26]. Although the definite demonstration of increased cardiovascular morbidity and mortality in PCOS women is still pending by now [27], cardiovascular risk factors cluster in these patients [28], and increased prevalence of coronary artery calcification [29], carotid atherosclerosis [30], and impairment in carotid viscoelastic properties [31] have been reported in PCOS. Therefore, the increase of serum IL-18 concentration in PCOS patients might be due to the presence of subclinical atherosclerosis in these women.

In summary, the serum IL18 level was increased in PCOS patientsl, and correlaed with IR, obesity and hyperandrogenism. Moreover, since IL18 was a proinflammatory cytokine and a strong risk marker for cardiovascular disease, whether we can use anti-inflammatory drug to reduce the serum level of IL18 and consequently decrease the incidence of PCOS, which may shred a new light on prevention or treatment of PCOS, but it is still need be further investigated based on large population.

## Conclusion

IL-18 level was increased in PCOS patients, and correlated with insulin resistance, obesity and hyperandrogenism.

## Competing interests

The authors declare that they have no competing interests.

## Authors' contributions

YY, JQ, RL and MZ-L developed the concept and designed the study. YY, JQ and RL participated in the study execution, analysed and interpreted the data and drafted the manuscript. JQ and MZ-L revised the manuscript for intellectual content. All authors read and approved the final manuscript.
